# An Equivocal Final Link – Quantitative Determination of the Thermodynamic Efficiency of ATP Hydrolysis – Sullies the Chain of Electric, Ionic, Mechanical and Metabolic Steps Underlying Cardiac Contraction

**DOI:** 10.3389/fphys.2020.00183

**Published:** 2020-03-31

**Authors:** Christopher John Barclay, Denis Scott Loiselle

**Affiliations:** ^1^Auckland Bioengineering Institute, The University of Auckland, Auckland, New Zealand; ^2^Department of Physiology, The University of Auckland, Auckland, New Zealand

**Keywords:** thermodynamics, cardiac muscle, metabolism, myothermia, stoichiometry of mitochondrial ATP production

## Abstract

Each beat of the heart completes the final step in a sequence of events commencing with electrical excitation-triggered release of Ca^2+^ from the sarcoplasmic reticulum which, in turn, triggers ATP-hydrolysis-dependent mechanical contraction. Given that *Thermodynamics* is inherently detail-independent, the heart can be thus be viewed as a mechanical pump – the generator of pressure that drives blood through the systemic and pulmonary circulations. The beat-to-beat pressure-volume work (W) of the heart is relatively straightforward to measure experimentally. Given an ability to measure, simultaneously, the accompanying heat production or oxygen consumption, it is trivial to calculate the *mechanical* efficiency: ε = W/ΔH where ΔH is the change of enthalpy: (W + Q), *Q* representing the accompanying production of heat. But it is much less straightforward to measure the thermodynamic efficiency: *η* = W/ΔG_*ATP*_, where ΔG_*ATP*_ signifies the Gibbs Free Energy of ATP hydrolysis. The difficulty arises because of uncertain quantification of the substrate-dependent yield of ATP - conveniently expressed as the P/O_2_ ratio. P/O_2_ ratios, originally (“classically”) inferred from thermal studies, have been considerably reduced over the past several decades by re-analysis of the stoichiometric coefficients separating sequential steps in the electron transport system – in particular, dropping the requirement that the coefficients have integer values. Since the early classical values are incompatible with the more recent estimates, we aim to probe this discrepancy with a view to its reconciliation. Our probe consists of a simple, thermodynamically constrained, algebraic model of cardiac mechano-energetics. Our analysis fails to reconcile recent and classical estimates of PO_2_ ratios; hence, we are left with a conundrum.

## Introduction

Until early in the 21st Century, the accepted P/O_2_ ratio for the oxidative phosphorylation of glucose had been 38 moles of ATP per 6 moles of molecular oxygen, yielding a P/O_2_ ratio of 6.3. The equivalent values for palmitate (the most prevalent saturated fatty acid in the daily diet of the heart (Taegtmeyer et al., 2016) had been 129 moles of ATP per 23 moles of molecular oxygen, yielding a P/O_2_ ratio of 5.6. We refer to these as “classical” estimates. Both were consistent with the existence of integer values of the stoichiometric ratios separating consecutive steps in the mitochondrial electron transport system. However, since that time, there has accumulated an extensive literature detailing the mitochondrial oxidation of glucose, in particular. It is now widely accepted, to the point of adoption in undergraduate textbooks [see, for example, Boron and Boulpaep (2009)], that the yield is considerably less than the classical value given above. Glucose oxidation is now thought to result in the generation of only 30 or 31, rather than 38, moles of ATP [for highly readable reviews, see Rich (2003) or Salway (2004). The comparable value for palmitate oxidation has been lowered from 129 to 104 (Salway, 2004)]. Since oxygen input (6 moles per mole of glucose, 24 moles per mole of palmitate) has remained unchanged, while putative ATP output has been reduced, the efficiency of cardiac *recovery metabolism* must necessarily have diminished.

Our use of the phrase “recovery metabolism” reflects the fact that the energy cost of a cardiac twitch comprises two conceptually distinct but temporally contiguous components: “initial metabolism” (I) and “recovery metabolism” (R). Initial metabolism comprises the biochemical events that occur nearly simultaneously with contraction: namely, the ATP hydrolysis-funded cycling of actin-myosin cross-bridges and ion pumps, and the rapid regeneration of ATP at the expense of a limited pool of PCr. Recovery metabolism reflects the reversal of the initial biochemical change: that is, the regeneration of PCr by ATP via oxidative phosphorylation of metabolic substrates in the mitochondria. Aerobic recovery metabolism is hence the exclusive domain of the mitochondria. Any contribution of non-mitochondrial recovery metabolism is quantitatively unimportant in myocardial tissues. Indeed, it has been recognized for over 60 years that lactate is readily metabolized by the heart (Griggs et al., 1966; Chapman and Gibbs, 1974; Drake-Holland et al., 1983).

In contrast to recovery metabolism, initial metabolism can readily be divided into two further components: activation and force development. Activation metabolism commences immediately prior to force development and continues throughout the contractile event; it funds sarcolemmal excitation and sarcoplasmic reticular Ca^2+^- cycling – the electrical and ionic events which, acting sequentially, achieve excitation-contraction coupling.

It has been a long-standing challenge [commencing, unsurprisingly, with early investigations by AV Hill and colleagues using skeletal muscle (Hill, 1911, 1949; Hartree and Hill, 1922, 1928)], with further refinement by Bugnard (1934), to determine the ratio (*r*) of recovery metabolism (R) to initial metabolism (I):

(Eq. 1) r=R/I

Whereas in amphibian skeletal muscle at 0°C, as typically utilized by Hill and colleagues, these two components are temporally distinct, such is not the case for cardiac muscle experiments performed between room temperature and body temperature, where other methods of separation, applicable to the thermometric technique, must be employed. Thus Mast et al. (1990), utilizing data previously published by Mast and Elzinga (1988), which had arisen from measurements of heat production by rabbit right-ventricular papillary muscles undergoing brief trains of isometric contractions at 20°C, developed a numerical correction procedure. This deconvolution procedure quantified recovery heat production that had occurred during the antecedent brief period of activity, in addition to the “pure” recovery heat observed during the subsequent exponential decline of muscle heat production to its supra-basal value.

It is important to emphasize that the separation of initial and recovery heat using the deconvolution technique was achieved by eliciting a brief train of twitches, with the accompanying heat production being recorded by rapid-response “flat-bed” thermopiles. What we now describe is a method that can be applied using data arising from steady-state contractions in a flow-through microcalorimeter (Taberner et al., 2005, 2011, 2018; Han et al., 2009; Johnston et al., 2015). The method, based on a straightforward algebraic model, yields estimates of the thermodynamic efficiencies of both cross-bridge cycling and mitochondrial ATP production, thereby allowing us to quantify the aforementioned difference between “classical” estimates of mitochondrial efficiency and more recent ones that admit non-integer mitochondrial stoichiometric coefficients.

In order to pursue that objective, we present a simple, thermodynamically consistent, algebraic model. The model aims to allow calculation of substrate-dependent P/O_2_ ratios, thereby permitting comparison with the current ratios detailed above.

## Methods and Results

Since no animals were used, this study is exempt from animal ethical considerations.

We commence by defining conceptually and experimentally distinct components of overall enthalpy production (ΔH_*O*_):

(Eq. 2) ΔH=OΔH+BΔH+AΔH+X-bΔH   mito

**FIGURE 1:**
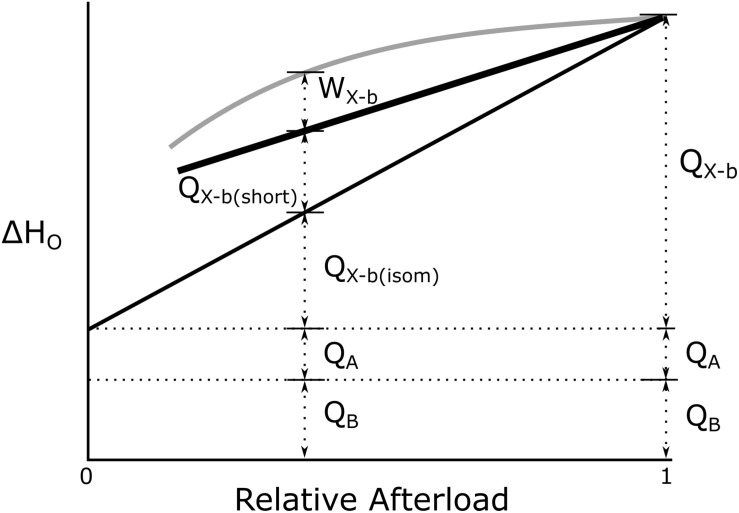
Conceptual basis of overall cardiac enthalpy output as a function of afterload. Q_*B*_ and Q_*A*_ define basal and activation heat, respectively. Heat from cross-bridge cycling in isolated cardiac muscle preparations arises from two distinct temporal phases: isometric [Q_*X–b(Isom*)_] and reduction of filament overlap [Q_*X–b(short*_)] corresponding, respectively, to the isovolumic and auxotonic phases of contraction of the heart *in vivo*. *W* denotes cross-bridge work. [Modified from Tran et al. (2017) under the aegis of the Rights Link^®^-Copyright Clearance Center; Account Number: 3000104389; License Number: 4678431442941].

The first three variables are the enthalpy outputs arising from basal metabolism, activation and cross-bridge cycling, respectively. The enthalpy from the first two of those appears entirely as heat (denoted “Q” in Figure 1) whereas enthalpy from cross-bridge cycling can appear as both heat and mechanical work.

The further separation of “isometric heat” [Q_*X–b*(*Isom*)_] and “shortening heat” [Q_X–b(*Short*)_] (Figure 1) is again purely conceptual, reflecting the fact that, in the beating heart, a period of isovolumic contraction necessarily precedes a period of auxotonic shortening. No fundamental difference of cross-bridge energetics between the isometric and shortening phases is implied.

We next capitalize on an experimentally straightforward simplification. It is trivial to “null” the basal enthalpy contribution (ΔH_B_; Eq. 2) electrically when making thermal measurements. When this is done, the magnitude of initial enthalpy production (ΔH_A_ + ΔH_X–b_) is revealed.

As foreshadowed above, initial enthalpy can be further separated into its underlying components, activation enthalpy and cross-bridge enthalpy, by use of a suitable inhibitor of cross-bridge cycling. This has recently been achieved in cardiac muscle by Tran et al. (2017) and by Pham et al. (2017) using the agent blebbistatin, the effectiveness of which had previously been demonstrated in skeletal muscle (Barclay, 2012). Blebbistatin was chosen because it: (i) achieves complete inhibition of cross-bridge turnover (Kovács et al., 2004; Allingham et al., 2005), (ii) does not affect excitation-contraction coupling (Farman et al., 2008), and (iii) does not affect the Ca^2+^-sensitivity of the contractile proteins (Dou et al., 2007). Using this cross-bridge inhibitor, Pham et al. (2017) found activation enthalpy to be both length-independent and force-independent (see Figure 1). These findings allow unambiguous quantification of ΔH_X–b_ at any given afterload. In the following, we focus on the afterload that maximizes cross-bridge efficiency since it too, is equally unambiguous.

Experimental quantification of cross-bridge heat (Q_X–b_) and cross-bridge work (W) defines cross-bridge enthalpy (δH_X–b_), thereby allowing definition of *macroscopic* cross-bridge efficiency (ε_X–b_):

(Eq. 3) $$\varepsilon_{X-b}=\frac{W}{W+Q_{X-b}}\equiv \frac{W}{\delta \,H_{x-b}}$$

where δH_*x–b*_ is the enthalpy change, as heat plus work, associated with cross-bridge cycling. It arises from the net breakdown of PCr, subsequent to the hydrolysis of ATP, which powers cross-bridge cycling, and the subsequent rapid buffering of ATP at the expense of PCr by the creatine kinase reaction, and is distinct from *overall* efficiency (ε_o_):

(Eq. 4) $$\varepsilon_{o}=\frac{W}{W+Q_{X-b}+Q_{\mathrm{mito}}}$$

**FIGURE 2:**
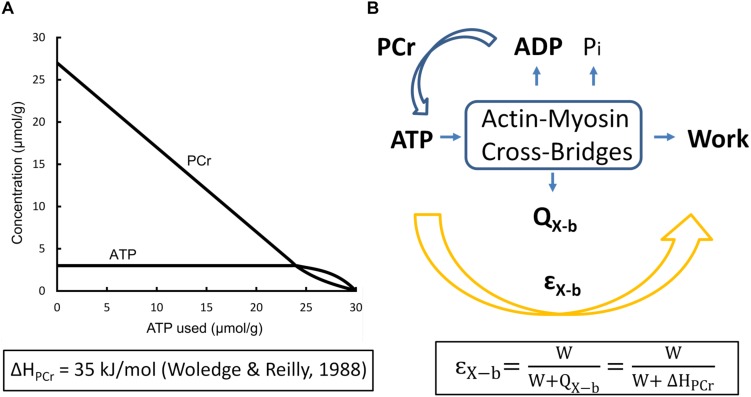
Events underlying cross-bridge metabolism. **(A)** The creatine-phosphokinase (Lohmann) reaction. Note the relative independence of ATP and PCr concentrations until the latter is nearly exhausted [based on Figure 4.2, Page 142 of Woledge et al. (1985)] **(B)** The Gibbs Free Energy of ATP hydrolysis as a function of the progressively increasing concentration of inorganic phosphate (Pi) in the absence of mitochondrial function, reflecting events shown in **(A)**: G_*ATP*_ = Δ*G^o^* + *RT*ln⁡([*ADP*][*Pi*]/[*ATP*]), where the Standard Gibbs Free Energy of ATP hydrolysis, $G_{A\,T\,P}^{o}=-30\,k\,J/m\,o\,l$, *R* is the Universal Gas Constant (8.31 kJ/mol), *T* is absolute temperature (310 K), and [ATP] and [ADP] are assumed to be 5 mmol/L and 1 μmol/L, respectively, under steady-state conditions of twitch force production. (Note the inverse scale on the ordinate).

The biochemical correlate of the expenditure of cross-bridge enthalpy is the production of ADP and Pi which, in the presence of PCr, regenerates ATP with rapid kinetics. But the concentration of PCr in cardiac myocytes is modest (of the order of 20–30 mmol L^–1^) so that work can be sustained only briefly from this source of ATP (see Figure 2A). That is, in the absence of recovery metabolism, cross-bridge cycling has but a brief existence.

Our aim is to estimate the *thermodynamic* efficiency of cross-bridge cycling (η_X–b_), which does not depend on the enthalpy of phosphocreatine, H_PCr_, but rather on the Gibbs Free Energy of ATP (ΔG_ATP_), a variable whose numeric value cannot be measured by thermometric or calorimetric means:

(Eq. 5) $$\eta_{X-b}\equiv \frac{W}{\Delta \,G_{\mathrm{ATP}}}=\frac{W}{\Delta \,H_{X-b}}\frac{\Delta \,H_{\mathrm{PCr}}}{\Delta \,G_{\mathrm{ATP}}}=\varepsilon_{\mathrm{Xb}}\frac{\Delta \,H_{\mathrm{PCr}}}{\Delta \,G_{\mathrm{ATP}}}$$

where ΔH_PCr_ and ΔG_ATP_ are molar values, and where

(Eq. 6) $$\Delta G_{\text{ATP}}=\Delta G_{\text{ATP}}^{o}+\mathrm{RT}\ln\left(\frac{\left[\mathrm{ADP}\right]\,\left[\mathrm{Pi}\right]}{\left[\mathrm{ATP}\right]}\right),$$

as shown in the Legend of Figure 2. We now capitalize on an insight due to Wilkie (1974):

(Eq. 7) η=oη⋅X-bη              m⁢i⁢to

where the subscript “o” again signifies “overall,” indicating the combined thermodynamic efficiencies of cross-bridge cycling and mitochondrial oxidative phosphorylation operating in series.

We next exploit a fortuitous *approximation*: the difference in magnitudes between ΔH and ΔG for the oxidative metabolism of each of carbohydrates, fatty acids and alcohol is negligible (Battley, 2002). The validity of this approximation is demonstrated in Table 1, where the rightmost column expresses the difference in magnitudes of enthalpy and Gibbs free energy, as a percentage of the former, for selected common metabolites.

**TABLE 1:** Difference between H^o^ and G^o^ for the oxidative metabolism of common metabolites at 25°C.

Metabolite	ΔH^o^ (kJ/mol)	ΔG^o^ (kJ/mol)	Percentage
Acetic acid	−874.54	−874.30	0.03
Glucose	−2803.03	−2881.26	–2.79
Ethanol	−1366.83	−1325.32	3.04
Glycerol	−1655.40	−1654.46	0.06
Sucrose	−5640.87	−5784.20	–2.54
Palmitic acid	−9977.83	−9789.70	1.89

*Data selected from Table 6 of Battley (2002). The right-most column expresses the percentage difference between the enthalpy and Gibbs free energy for selected metabolites at 298.15 K.*

Since the average difference of the entries in the right-most column of Table 1 is −0.05%, it is evident that Δ*G*_*S*_ ≈ Δ*H*_*S*_, where the subscript “S” denotes “substrate.” Hence, Eq. 7 can be re-expressed as:

(Eq. 8) $$\eta_{o}=\eta_{X-b}\eta_{m\,i\,t\,o}or\eta_{m\,i\,t\,o}\approx \;\frac{\varepsilon_{o}}{\eta_{X-b}}$$

where ε_*o*_ signifies *macroscopic* overall efficiency.

(Eq. 9) $$\text{But}\,\eta_{X-b}=\frac{\Delta \,H_{P\,C\,r}}{\Delta \,G_{A\,T\,P}}\,\varepsilon_{X-b}\&\eta_{m\,i\,t\,o}=\frac{\varepsilon_{o}}{\varepsilon_{X-b}}\,\frac{\Delta \,G_{A\,T\,P}}{\Delta \,H_{P\,C\,r}}$$

$$\text{so}\eta_{X\,b}=\frac{\varepsilon_{X-b}}{\varepsilon_{o}}\frac{\Delta \,H_{X-b}+H_{m\,i\,t\,o}}{\Delta \,H_{X-b}}$$

(Eq. 10) $$\text{thus}\,\frac{\varepsilon_{X-b}}{\varepsilon_{o}}=1+\frac{\Delta \,H_{m\,i\,t\,o}}{\Delta \,H_{X-b}}=1+r,s\,i\,n\,c\,e\,r=\frac{\Delta \,{\text{H}}_{\text{mito}}}{\Delta \,{\text{H}}_{\text{X}-\text{b}}}$$

(Eq. 11) $$\text{Hence,}\eta_{\text{mito}}\;\frac{1}{1+r}\frac{\Delta \,G_{A\,T\,P}}{\Delta \,H_{P\,C\,r}}$$

It is clear from Eq. 11 that estimation of the thermodynamic efficiency of recovery metabolism requires numeric estimates of *r*, G_ATP_, and H_PCr_. Very few estimates of *r* arising from experiments on cardiac muscle have been published. But those of which we are aware have all utilized the method developed by Woledge and described above (Mast et al., 1990). Using this technique during either single twitches or trains of ten twitches at a stimulation rate of 0.2 Hz in rabbit right-ventricular papillary muscles, these authors found the value of *r* to be 1.18 at 20°C. Using comparable techniques on mouse left-ventricular papillary muscles undergoing isovelocity contractions at 30°C, values of 1.16 Barclay et al. (2003) and 1.20 (Barclay and Widén, 2010) have subsequently been reported. Given the closeness of these three independent estimates, we have adopted their average value: *r* = 1.18.

The stoichiometry of ATP synthesis from PCr hydrolysis, is 1:1 and the best estimate of its enthalpy output, achieved using microcalorimetry, with both acid hydrolysis and enzymatic hydrolysis of PCr, is 35 kJ mol^–1^ (Woledge and Reilly, 1988).

Our best estimate of the Gibbs Free Energy of ATP hydrolysis, under conditions prevailing in the myocardium, probably remains that of Kammermeier et al. (1982): 60 kJ mol^–1^, arising from experiments performed on isolated, perfused, electrically paced female Sprague-Dawley rat hearts subjected to biochemical analyses of high-energy phosphates following rapid freezing. This early value remains in remarkable agreement with the more recent determination of 59.7 kJ mol^–1^, arising from *in situ* ATP flux measurements recorded in 17 healthy human hearts of either sex using the technique of magnetic resonance spectroscopy (Weiss et al., 2005).

Given these three required parameter values, Eq. 11 immediately returns:

(Eq. 12) $$\eta_{\text{R}}\approx \frac{1}{1+1.18}\cdot \frac{\frac{60\,k\,J}{m\,o\,l}}{\frac{35\,k\,J}{m\,o\,l}}\approx 0.786$$

**FIGURE 3:**
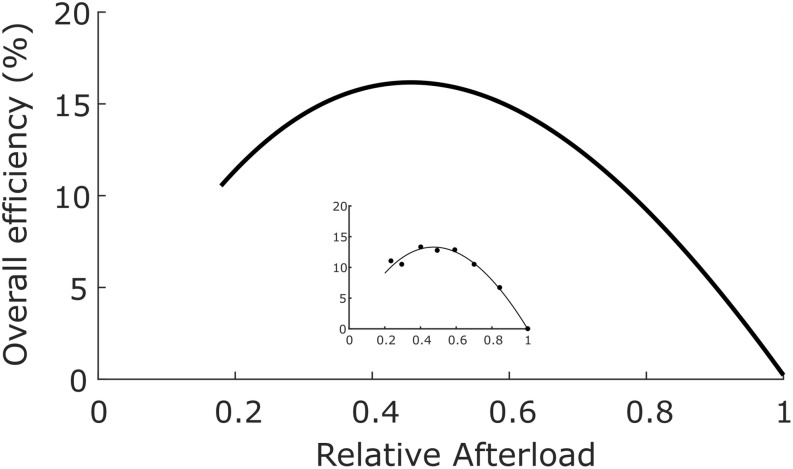
Overall efficiency as a function of relative afterload. Average data from 56 rat left-ventricular trabecula at 32°C. Peak overall efficiency (0.155) occurred at a relative afterload of 0.45. Inset: data from a single representative trabecula; data fitted by cubic regression. [Figure modified from Tran et al. (2016) under the aegis of the Rights Link^®^-Copyright Clearance Center; *Permission received from The American Physiological Society*].

In order to calculate cross-bridge thermodynamic efficiency, we turn to data published by Tran et al. (2016) arising from experiments performed at 32°C using left-ventricular trabecula from Dahl salt-sensitive rats and their congenic controls. These authors apportioned 56 animals into four equal-size cohorts fed on either high- or low-salt diets. Since there were no differences of efficiency among the four groups, the results were averaged and displayed in Figure 3, where the mean peak value of ε_X–b_ ± SEM was found to be 0.155 ± 0.059.

Given that η_X–b_ = ε_o_/η_mito_ (Eq. 8), it follows that the thermodynamic efficiency of cross-bridge work performance, (η_X–b_), is 0.155/0.786 or 0.20.

**FIGURE 4:**
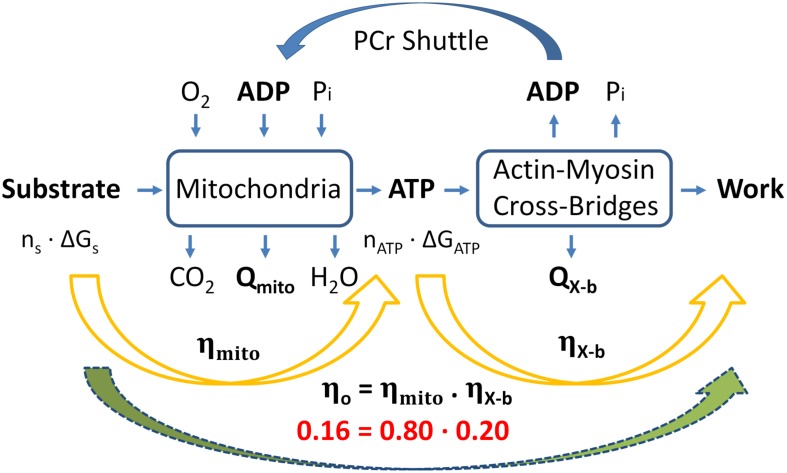
Summary of overall “classical” thermodynamics of cardiac energetics. The right-hand orange arrow denotes the production of ATP via the Lohman reaction, which comprises cross-bridge enthalpy production (as shown in Figure 2B). The left-hand orange arrow denotes mitochondrial metabolism in which *n* moles of metabolic substrate are oxidizsed to provide sufficient Gibbs free energy to produce *n* moles of ATP to fund cross-bridge cycling. Overall thermodynamic efficiency (0.16) is calculated as the product of mitochondrial and cross-bridge thermodynamic efficiencies; 0.8 and 0.2, respectively. [Modified from Barclay (2017) under the aegis of Rights Link^®^-Copyright Clearance Center: permission received from *John Wiley and Sons*].

With estimates provided for the thermodynamic efficiencies of both cross-bridge energy expenditure (0.20, Figure 3) and mitochondrial energy expenditure (0.79, Eq. 12), we present a pictorial summary of overall thermodynamic efficiencies of cross-bridge cycling and resulting mitochondrial oxidative phosphorylation in Figure 4.

## Discussion

In order to focus on the uncertain link in the chain of events commencing with excitation-contraction and culminating with metabolic restoration of ATP, we have developed a simple and straightforward algebraic model. The model enables the separation and quantification of the thermodynamic efficiencies of cross-bridge cycling and attendant mitochondrial oxidative phosphorylation.

### Critique of the Model

Whereas it contains no assumptions, the model does exploit two approximations: (i) the ratio of heat produced by the mitochondria to heat generated by cross-bridge cycling is 1.18 (the mean of three values reported in the Literature) and (ii) the quantitative differences between the enthalpies and Gibbs free energies of oxidation of common metabolic substrates is sufficiently small that they may be ignored (Table 1). Furthermore, with a single exception (Weiss et al., 2005), its parameter values gleaned from the literature pertain to rodent (rat or mouse) myocardial tissues. With these caveats, we conclude that the maximum thermodynamic efficiency of work generation by the myocardium (η_*o*_) is a modest 16%. This implies (see Figure 4) that cross-bridges convert 20% of the free energy from ATP hydrolysis into work and mitochondrial oxidation transfers 80% of the free energy available in metabolic substrates into free energy in the form of ATP.

It is important to stress that the value of 20% efficiency is restricted to *mechanical* (i.e., cross-bridge) performance only. As shown schematically in Figure 1, it explicitly excludes both the basal and activation components. Inclusion of these two “overhead” metabolic costs, neither of which *directly* funds cross-bridge cycling, would, of course, reduce the overall thermodynamic efficiency of the heart even further – perhaps by as much as 40–50%. Nevertheless, our finding of 16% for the overall efficiency of cross-bridge cycling (ε_*o*_) aligns well with the seminal values reported by Gibbs et al. (1967) for rabbit right-ventricular papillary muscles: 16% to 19%, and by Neely et al. (1967) for isolated, perfused, rat whole-heart preparations: 16% to 17%. Note that both of these estimates differ greatly from the “isoefficiency” value of 40% promoted by Khalafbeigui et al. (1979), Suga et al. (1980, 1981, 1986), Suga (1979, 1990), but which was based on a conceptual error, as revealed by Han et al. (2012).

### Error Analysis of the Model

A rigorous examination of the susceptibility of a numeric model to the value of its parameters can be achieved through a formal Error Analysis. Appendix 1 provides the relevant derivation and shows a small sample of results. Since there is a total of five input parameters [denoted by subscripted values of putative fractional errors (*f*), each of which has only a single constraint (*f* ≥ 0)], an infinite number of Fractional Errors of Measurement are available to be modeled. Of the five input parameters, *f*_*PCr*_ may be the most likely to be in error given that its nominal value (35 kJ/mol) was obtained using a bicarbonate buffer (Woledge and Reilly, 1988), prior to the discovery of Na^+^-HCO_3_^–^ exchangers resident in the sarcolemmal membranes of cardiac myocytes, However, with respect to any potential Error of Estimate arising from this source, the authors of the study state: “*In this solution HCO_3_^–^ will provide approximately 17% of the internal buffer capacity. This will have the effect of increasing the value of –ΔH_*ob*_, by 1 kJ mol^–1^*” - or an uncertainty of less than 3% in that parameter.

Furthermore, as shown in Appendix Figure A1(A), even in a worst-case scenario, with Errors of Measurement of twice the magnitude given above in *each* of *r*, ΔG_*ATP*_, and ΔH_*PCr*_, the Error of Estimate in *R* would be unlikely to exceed 10%, This would translate to an ATP yield of 35 mol_*ATP*_ per mol_*glucose*_, still considerably greater than recent estimates arising from mitochondrial experiments, thereby again underscoring the “classical-mitochondrial” difference.

Appendix Figure A1(B) shows that the Error of Estimate of Initial Metabolism (I) is comparatively insensitive to errors of estimate of its input parameters (Work and Heat).

### Relative Insensitivity of ε_*o*_ to Hypertension

Using the microcalorimetric technique, no difference has been found in overall cross-bridge efficiency between trabecula isolated from left and right ventricles of *healthy* rats at either room temperature (Han et al., 2013) or body temperature (Pham et al., 2017). These results in healthy cardiac tissues have been largely duplicated in heart failure models. Thus Han et al. (2014) found no difference of ε_*o*_ between trabecula dissected from the left ventricles of streptozoticin-induced Type I diabetic rats and their untreated controls. Nor were differences revealed among trabecula from Dahl salt-sensitive rats and their congenic controls, whether fed low- or high-salt diets (Tran et al., 2016). Similar results obtained in trabecula from both ventricles of hearts in which pulmonary arterial hypertension had been induced by injection of monocrotaline and untreated control trabecula (Pham et al., 2018). Hence, in each of these models overall cardiac function was compromised but the energetics of cycling cross-bridges and the associated mitochondrial energy supply were unaffected. In fact, the only hypertension-sensitive difference appeared in trabecula from spontaneously hypertensive (SHR) animals where overall cross-bridge efficiency was lower in trabecula from both failing and non-failing hearts than in those of age-matched control animals. Whereas this list is far from comprehensive, the contrast between the results of a naturally arising model (SHR), and those of three unrelated experimentally induced heart failure models, is intriguing.

### Entropy Production

The modest value of thermodynamic efficiency of cross-bridge cycling (20%) shown in Figure 4 implies a high rate of entropy production. This implication is qualitatively consistent with the conceptual “energy well” picture proffered by TL Hill and colleagues (Hill, 1974; Eisenberg and Hill, 1979; Eisenberg et al., 1980), and subsequently exploited by Barclay (1999). Under any of these authors’ formulations, potential Gibbs Free Energy of ATP hydrolysis remains necessarily unused whenever a cross-bridge either attaches belatedly or detaches prematurely, rather than traversing the full descent of its parabolic Free Energy profile. Hence, by the Second Law: -ΔH_*ATP*_ = ΔG_*ATP*_ + TΔS_*ATP*_, the entropy (ΔS) so produced cannot subsequently be exchanged (Chapman and Loiselle, 2016), thereby demonstrating that the hydrolysis of an ATP molecule by actomyosin (Figure 2B) is an irreversible process – a conclusion reached earlier by use of a thermodynamically constrained mathematical model of the cross-bridge cycle (Loiselle et al., 2010).

In contrast to the production of entropy by cross-bridge cycling, our model predicts that the extent of inefficiency attributable to mitochondrial oxidative phosphorylation of metabolic substrates is modest; its primary source is likely to be leakage of protons back across the inner mitochondrial membrane, without contributing to ATP production via the electron transport system. An early calculation (Loiselle, 1987) suggested that this source may contribute upward of 5 mW g^–1^ to the basal component of total enthalpy production (Figure 1). This speculative result has subsequently been extensively investigated by Brand et al. (1994) who concluded that the increased rates of oxygen consumption at high proton motive force could be attributed to this source and that it further contributes to the basal metabolic rate of homeotherms (Brand, 1990; Porter and Brand, 1993; Rolfe and Brand, 1996).

### Mitochondrial P/O_2_ Ratios

The prediction of a modest extent (20%) of inefficiency attributable to recovery metabolism warrants further investigation. We commence by noting that, given any value of *r* (the ratio of recovery metabolism to initial metabolism, Eq. 1), then the P/O_2_ ratio, *p*, can be calculated as:

(Eq. 13) $$p=\frac{\Delta \,H_{S}}{\Delta \,H_{P\,C\,r}\,\left(r+1\right)}$$

where ΔH_*S*_ is the substrate enthalpy per mole of oxygen consumption. Early (mid-20th Century) experiments returned values for *p* in the vicinity of *6.3* for NADH-linked metabolites [for a comprehensive Review, see Table 1 of Hinkle (2005)]. This is the value that we previously labeled “classical.” As detailed above, with glucose as substrate, it generates a stoichiometric ratio of some 37–38 molecules of ATP per mole of O_2_. With palmitate as substrate, the classical value is 129 molecules of ATP per mole of O_2_. The equivalent current values are 30 and 104, respectively.

What value of *p* is consistent with our estimate of η_*mito*_? If η_*mito*_ = 0.8, then 80% of ΔH_*S*_ is transferred to ΔG in ATP. In that case, then the ATP yield, per mole of glucose, would be 2802 × 0.8/60 or 37.3. This value is consistent with the classical, rather than recent, estimates of P/O_2_ ratios. Is it possible that discrepancies of this magnitude prevailed in the muscles considered in the current investigation but were obscured by experimental uncertainties? One way in which η_*ATP*_ could be overestimated is if the assumed value of ΔG_*ATP*_ were too low. However, if n_*ATP*_ were 30 instead of 38, then ΔG_*ATP*_ would have to be an unrealistically high 76 kJ mol^–1^ to account for η_*mito*_ of 0.8. Recall (see Results) its re-measured and, re-confirmed, value of 59.7 kJ mol^–1^ in human hearts *in situ* (Weiss et al., 2005).

A second factor to consider (see Eq. 13) is the substrate enthalpies. Might the classical value (2800 kJ mol^–1^) for glucose have been overestimated? Using the technique of adiabatic calorimetry, Kabo et al. (2013) found the enthalpy of α-D-glucose to be 2802.4 kJ mol^–1^. This value is in remarkable accord with that of 2803.03 kJ mol^–1^ calculated by Battley (2002). Thus, if the Gibbs Free Energy of cytoplasmic ATP is 60 kJ mol^–1^, there would be sufficient energy to generate 2803 × 0.8/60 or 37.4 ATP molecules per mole of glucose – a value consistent with the theoretical limit of 37–38.

Comparable stoichiometric concerns obtain for the mitochondrial oxidation of palmitate. Salway (2004) details how the classical value of 129 molecules of ATP per mole of palmitate reduces to a value of 104 when non-integral values for intermediate steps in the mitochondrial sequence are allowed. However, Levine et al. (2014), using the technique of differential scanning calorimetry, reported the enthalpy of oxidation of methyl palmitate to be 10694 kJ mol^–1^. Correction for the presence of the methyl group, and multiplication by 0.8/60 (as above), would yield 142.6 ATP per mole, a value readily accommodating the theoretical maximum of 129 molecules of ATP per mole.

**FIGURE 5:**
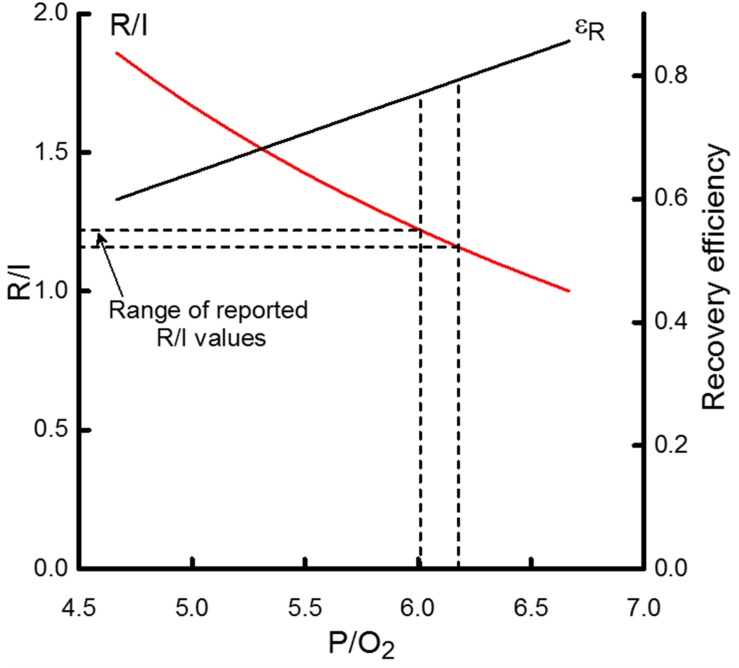
The dependence on the P/O_2_ ratio of the ratio of recovery heat to initial heat (R/I) and recovery efficiency (ε_*R*_). R/I values are scaled by the left-hand *y*-axis and ε_*R*_ by the right-hand *y*- axis. The horizontal dashed lines indicate the range of reported R/I values for cardiac muscle; that range corresponds to P/O_2_ values from approximately 6 to 6.2, indicated by the vertical dashed lines. That range of P/O_2_ values, in turn, is consistent with ε_*R*_ values of 0.77 to 0.79, as indicated by the intersection of the vertical dashed lines and the curve relating the P/O_2_ dependence of ε_*R*_. If ATP yield were approximately 30 ATP/glucose, then P/O_2_ would be 5 which would correspond to R/I and ε_*R*_ values of 1.7 and 0.64, respectively.

The third factor with potential to affect the estimated ATP yield is the value of *r*. If n_*ATP*_ were actually 30 instead of 38 for glucose oxidation, then the amount of substrate oxidized, and recovery heat produced, would have to be 38/30 or 1.27–times greater than assumed by acceptance of the average measured value of *r* = 1.18. That is, *r* would have to be 1.52, a value that greatly exceeds the upper 95% confidence limit arising from its experimental determinations of ∼1.22 (Barclay et al., 2003). The consequences of changed values of any of these three factors are displayed in Figure 5.

What could bring clarity to these disparities would be a study designed to investigate the issue by simultaneous measurement of heat production and oxygen consumption. Fortunately, such a study has been performed, albeit in skeletal muscle. Nevertheless, given the similarity of the cellular machinery between cardiac and skeletal muscles, we turn attention to the results of Mahler (1979) who, using Sartorius muscles from *Rana pipiens*.” at 20°C, compared the suprabasal rate of oxygen consumption during recovery from brief tetani (0.2, 0.5, and 1.0 s) with the amount of ATP hydrolyzed (measured indirectly as the decrement of PCr concentration) during the same period. The mean ratio of the PCr decrement to the amount of suprabasal oxygen consumed, subsequently shown to obey first-order kinetics (Mahler, 1985), thereby simplifying interpretation, averaged over a total of 62 tetani, was 6.58 ± 0.55 – consistent with the “classical” P/O_2_ value of 6.3.

In support of this convincing finding, Lou et al. (2000) made simultaneous measurements (at 19°C) of heat production and oxygen consumption, measured polarographically, during metabolic recovery of 10 bundles of white fibers from the dogfish. Their reported value for recovery metabolism of 84% in these skeletal muscle preparations echoes ours of 80% in cardiac muscle – in accord with the “classical” values, but again at variance with more recent estimates that yield non-integer stoichiometric coefficients.

Non-integer stoichiometric coefficients are most commonly attributed to “proton leakage.” Mazat et al. (2013) provide a comprehensive discussion of this issue, emphasizing especially passive proton leak (a consequence of non-zero membrane conductance), and the role of uncoupling proteins, but considering also “slip” or “intrinsic uncoupling” as a consequence of a decrease in the efficiency of proton pumps. In a similar vein, van der Zwaard (2016) offer the timely reminder that mitochondrial inhibition may be caused by either nitric oxide production or by “methodological issues related to permeabilizing procedures or isolation.” The latter, of course, is not limited to mitochondrial isolation; cardiac preparations (especially minute trabecula) can likewise be unwittingly damaged during isolation. In any case it does not seem to be unreasonable to suggest that the higher temperature at which mitochondrial experiments are conducted [37°C; see, for example (Pham et al., 2014) and accompanying commentary (Patel and McDonough, 2014)] *vis-à-vis* thermometric experiments (20–30°C; see section “Introduction”) may have the unintended result that proton leakage is maximized under the former condition, thereby contributing to the disparity between “classical” and “mitochondrial” estimates of thermodynamic efficiency of cross-bridge cycling.

### An Unresolved Issue Underling Mathematical Modeling of Cross-Bridge Energetics

In order for any mathematical model (independent of its complexity) in which the cross-bridge passes through a series of states from “unattached” to “detached” to be thermodynamically constrained, it must obey the following relation (Hill, 1989):

(Eq. 14) $$\frac{\prod_{i}f^{+}}{\prod_{i}f^{-}}=e^{\Delta \,G_{A\,T\,P}/R\,T}$$

That is, the ratio of the product of all forward reactions (*f*^+^) to the product of all reverse reactions (*f*^–^) is constrained by the Gibbs Free Energy of ATP hydrolysis (see Eq. 6). But despite the results of Mahler (1985) and Lou et al. (2000) there remains no agreement between the “Classical” and “Mitochondrial” values of ΔG_*ATP*_. As noted above, if n_*ATP*_ were 30 (in concert with modern “Mitochondrial” estimates) instead of 38, then ΔG_*ATP*_ would have to be an unrealistically high 76 kJ mol^–1^ (instead of the “Classical” value of 60 kJ/mol, in order to account for η_*mito*_ of 0.8 as predicted by our simple model (Figure 4). Clearly, the yawning difference between these two estimates of n_*ATP*_ and, consequently, the numeric value of ΔG_*ATP*_ casts uncertainty on the accuracy of all mathematical models of the cross-bridge cycle.

### Summary

Using a simple algebraic model, containing only three parameters (*r*, ΔH_*PCr*_, and ΔG_*ATP*_), each of which has been experimentally well-attested, we find that the thermodynamic efficiency of cross-bridge cycling is 20%, while that of mitochondrial oxidative phosphorylation is 80%, giving a value of 16% for overall thermodynamic efficiency of the mechanical activity of the heart. We show that the latter value is consistent with those measured in experiments undertaken using flow-through microcalorimetry. Nevertheless, we are left with a biophysical-biochemical conundrum. We are unable to reconcile the discrepancy between “Classical” (i.e., thermodynamically constrained) and “Mitochondrial” (i.e., stoichiometrically unconstrained) P/O_2_ ratios – a situation that prevents full thermodynamic understanding of the cascade of events comprising the cardiac twitch.

## Data Availability Statement

The datasets generated for this study are available on request to the corresponding author.

## Author Contributions

CB and DL contributed to the conception and design of the work, acquisition, analysis, interpretation of the data, drafting the manuscript, approving the final version of the manuscript, and agreed to be accountable for all aspects of the work in ensuring that questions related to the accuracy or integrity of any part of the work have been appropriately investigated and resolved, and all persons designated as authors qualify for authorship, while all those who qualify for authorship are listed.

## Conflict of Interest

The authors declare that the research was conducted in the absence of any commercial or financial relationships that could be construed as a potential conflict of interest.

## Figures and Tables

**APPENDIX A1:**
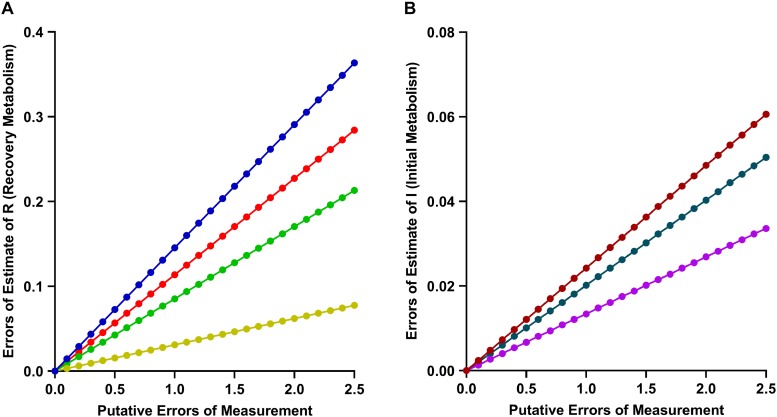
Relative Errors of Estimate of Recovery Metabolism (R). **(A)** Relative error in Recovery Metabolism attributable singly to *r* (red), ΔG_*ATP*_ (green), ΔH_*PCr*_ (orange) and collectively to all three input terms (blue). **(B)** Relative error in Initial Metabolism attributable to Work (purple), Heat (black) and collectively to both (red).

## Appendix

It is required to determine the Errors of Estimate of *R* and *I*. To that end, we define *f* as the fractional error in each component of *R* and *I*. For example, if *f* = 0.1, then the measurement error of ΔH_*PCr*_ is expressed as 48 ± 4.8 J mol^–1^.

The Error of Estimate of η_*R*_ is given by:

$$\sqrt{\begin{array}{c} {\left(\frac{\partial \eta_{R}}{\partial r}\,f\cdot r\right)}^{2}+{\left(\frac{\partial \eta_{R}}{\partial \Delta \,G_{A\,T\,P}}\,f\cdot \Delta \,G_{A\,T\,P}\right)}^{2}+{\left(\frac{\partial \eta_{R}}{\partial \Delta \,H_{P\,C\,r}}\,f\cdot \Delta \,H_{P\,C\,r}\right)}^{2} \end{array}}$$

where the nominal values of *r*, ΔG_*ATP*_, and ΔH_*PCr*_ are 1.2, 60, and 48, respectively (see Text).

The Error of Estimate of η_*I*_ is given by:

$$\sqrt{{\left(\frac{\partial \eta_{I}}{\partial W}\,f\cdot W\right)}^{2}+{\left(\frac{\partial \eta_{I}}{\partial Q}\,f\cdot Q\right)}^{2}}$$

where the numeric values of W and Q arise from the original data used to construct Figure 3 and are 1.4625 and 7.6780, respectively (Tran et al., 2016).

## Abbreviations

Glossary of symbols: Δ G_*ATP*_: Gibbs free energy of ATP hydrolysis
Δ H: change of enthalpy: (W + Q)
H_*A*_: enthalpy of activating contraction
H_*B*_: enthalpy of basal metabolism
H_*X–b*_: enthalpy arising from cross-bridge cycling
H_*mito*_: enthalpy of mitochondrial oxidative phosphorylation
Δ H_*PCr*_: molar enthalpy change of creatine phosphate hydrolysis
δ H_*x–b*_: extent of PCr breakdown by cross-bridges x molar enthalpy change
W/ Δ H: W/(W + Q): ε mechanical efficiency
W/ Δ G: η thermodynamic efficiency
I: initial metabolism
Q: heat
Q_*X–b*_: heat arising from cross-bridge cycling
Q_*X–b(Isom)*_: heat arising from X-b cycling during the isometric phase of an auxotonic contraction
Q_*X–b(Short)*_: heat arising from X-b cycling during the shortening phase of an auxotonic contraction
R: recovery metabolism
r: ratio of recovery metabolism to initial metabolism: R/I
W: work
X-b: cross-bridge.
